# Why a distinct medical stream is necessary to support patients using cannabis for medical purposes

**DOI:** 10.1186/s42238-023-00195-8

**Published:** 2023-07-04

**Authors:** Cecilia Costiniuk, Caroline A. MacCallum, Michael Boivin, Sergio Rueda, Gary Lacasse, Zach Walsh, Paul J. Daeninck, Shari Margolese, Enrico Mandarino, Jagpaul Kaur Deol, Tatiana Sanchez, Alan D. Bell

**Affiliations:** 1 Chronic Viral Illness Service/Division of Infectious Diseases, McGill University Health Centre, McGill Cannabis Research Centre and Research Institute of the McGill University Health Centre, Montreal, QC H4A 3J1 Canada; 2 Department of Medicine and Division of Palliative Care, University of British Columbia and Greenleaf Medical Clinic, Vancouver, BC Canada; 3 CommPharm Consulting Inc., Barrie, ON Canada; 4 Department of Psychiatry and Institute of Health Policy, Management and Evaluation, Centre for Addiction and Mental Health, Institute for Mental Health Policy Research and Campbell Family Mental Health Research Institute, Temerty Faculty of Medicine, University of Toronto, Toronto, ON Canada; 5 Canadian AIDS Society, Ottawa, ON Canada; 6 Department of Psychology, University of British Columbia, Kelowna, BC Canada; 7 Department of Internal Medicine, CancerCare Manitoba, Max Rady College of Medicine, University of Manitoba, Winnipeg, Canada; 8 Canadian Institutes of Health Research (CIHR) Canadian HIV Trials Network, Vancouver, BC Canada; 9 MJardin Canada, Toronto, ON Canada; 10 Faculty of Pharmaceutical Sciences, University of British Columbia, Vancouver, BC Canada; 11 Department of Family and Community Medicine, University of Toronto, Toronto, ON Canada

**Keywords:** Cannabis, Cannabinoid-based medicine, Medical cannabis, Access

## Abstract

**Background:**

Since 2001, Canadians have been able to obtain cannabis for medical purposes, initially through the Access to Cannabis for Medical Purposes Regulations (ACMPR). The Cannabis Act (Bill C-45) came into force on October 17, 2018, replacing the ACMPR. The Cannabis Act enables Canadians to possess cannabis purchased from a licensed retailer without authorization for either medical or nonmedical purposes. The Cannabis Act is currently the guiding legislation which governs both medical and nonmedical access. The Cannabis Act contains some improvements for patients but is essentially the same as its previous legislation. Beginning in October 2022, the federal government is conducting a review of the Cannabis Act and is questioning whether a distinct medical cannabis stream is still required, given the ease of access to cannabis and cannabis products. Although there is overlap in the reasons for medical and recreational cannabis use, the distinct legislation of medical versus recreational use of cannabis in Canada may be under threat.

**Main body:**

A large segment of the medical, academic, research, and lay communities agree that there is a need for distinct medical and recreational cannabis streams. Perhaps most importantly, separation of these streams is necessary to ensure that both medical cannabis patients and healthcare providers receive the required support needed to optimize benefits while minimizing risks associated with medical cannabis use. Preservation of distinct medical and recreational streams can help to ensure that needs of different stakeholders are met. For example, patients require guidance in the form of assessing the appropriateness of cannabis use, selection of appropriate products and dosage forms, dosing titration, screening for drug interactions, and safety monitoring. Healthcare providers require access to undergraduate and continuing health education as well as support from their professional organizations to ensure medical cannabis is appropriately prescribed. Although there are challenges in conducing research, as motives for cannabis use frequently straddle boundaries between medical versus recreational cannabis use, maintenance of a distinct medical stream is also necessary to ensure adequate supply of cannabis products appropriate for medical use, to reduce stigma associated with cannabis in both patients and providers, to help enable reimbursement for patients, to facilitate removal of taxation on cannabis used for medical purposes, and to promote research on all aspects of medical cannabis.

**Conclusion:**

Cannabis products for medical and recreational purposes have different objectives and needs, requiring different methods of distribution, access, and monitoring. HCPs, patients, and the commercial cannabis industry would serve Canadians well to continue to advocate to policy makers to ensure the continued existence of two distinct streams and must strive to make ongoing improvements to the current programs.

## Background

In 2001, the Marijuana for Medical Purposes Regulations (MMPR), which has since evolved into the Access to Cannabis for Medical Purposes Regulations (ACMPR), was introduced, allowing individuals to obtain cannabis and cannabis products for the management of medical symptoms and diseases (Health Canada 2005). The ACMPR ensured that individuals can legally possess cannabis providing they have clinician authorization (Health Canada 2005). On October 17, 2018, the Cannabis Act (Bill C-45) came into force, replacing ACMPR regulations. These new regulations enable Canadians to possess medical or nonmedical cannabis purchased from a licensed retailer without authorization (House of Commons of Canada 2022). The overarching goals of this legislation were to eliminate the illicit cannabis market, to provide a safe regulated supply, and to prevent youth from accessing cannabis (House of Commons of Canada 2022). Beginning October 2022, the federal government is conducting a review of the Cannabis Act and is questioning whether a distinct medical stream is still required, given the ease of access to cannabis and cannabis products. As seen in Uruguay and certain US states, where cannabis has also been legalized, there is debate surrounding the best policy approach to cannabis use (Kilmer 2019). In a systematic review on international perspectives regarding implications of cannabis legalization, Bahji et al. identified 36 studies which explored various aspects of cannabis legalization including health, epidemiology, health service utilization, public policy, crime, and economic implications (Bahji and Stephenson 2019). The majority of studies reported that cannabis use increased over time, and there were increases in the number and rates of emergency room visits for cannabis-related presentations, such as cannabis intoxication and cannabis-related hyperemesis; however, reductions in the rates of opioid prescribing were also noted. Rates of major crime have also fallen by 15–30% in US states with legalized cannabis (Bahji and Stephenson 2019). There remains significant controversy regarding the overall impact of legalization, especially on mental health and public policy (Bahji and Stephenson 2019). Bahji et al. concluded that there is a paucity of findings related to cannabis legalization, and available studies are relatively heterogeneous, making conclusions difficult to draw at this time (Bahji and Stephenson 2019).

### What are the pros and cons of the current medical system?

The current medical access program for cannabis has both strengths and limitations. Advantages of legalization of nonmedical cannabis include reducing harms from criminalization of cannabis possession, regulating the distribution and content of product, and increasing government revenue through taxation. However, one of the most important limitations is that the Cannabis Act does not ensure medical cannabis patients will obtain any healthcare provider support as the act does not require this interaction. The Cannabis Act should provide mechanisms to support both patients and healthcare providers. Patients using cannabis for medical purposes require reliable sources of information and guidance from well-trained healthcare providers. Similarly, healthcare providers require ongoing support such as continuing education programs and appropriate documents for authorizing cannabis. Although some resources currently exist for healthcare providers and the public including information provided on websites by the government of Canada and programs created by universities, they are sparse and inadequate (Health Canada 2018; UBC CPD 2020).

Beyond direct patient benefits, medical involvement has contributed to the discovery of new therapeutic applications for cannabinoids including chronic pain management, multiple sclerosis, chemotherapy-induced nausea and vomiting, epilepsy, and palliative care, in patients who are insufficiently managed via other modalities. As such, cannabinoids are generally a highly acceptable option for many patients and may play a useful role as an adjunct therapy (Busse et al. 2021; Wang et al. 2021; Health Canada n.d.). In some reports, over half of cannabis consumers were able to reduce or adjust their use of other medications, including opioids, through addition of medical cannabis to their regimens (Minhas and Lunn 2022). In a systematic review, Nielsen et al. identified 19 preclinical and 9 clinical studies which provided evidence of an opioid-sparing effect of cannabinoids (Nielsen et al. 2017). Of the 19 preclinical studies, 17 provided evidence of synergistic effects from cannabinoids and opioid co-administration (Nielsen et al. 2017).

In general, randomized clinical trials provide stronger evidence than do observational studies, while rigorous observational studies provide stronger evidence than uncontrolled case series. Indications approved by national regulatory authorities, and for which they document conclusive or substantive evidence in a 2017 publication, includes chemotherapy-induced nausea and vomiting, patient-reported multiple sclerosis spasticity symptoms, and chronic neuropathic pain (National Academies of Sciences, Engineering, and Medicine, Health and Medicine Division, Board on Population Health and Public Health Practice, Committee on the Health Effects of Marijuana: An Evidence Review and Research Agenda 2017). Authors report moderate evidence for improving short-term sleep outcomes in individuals with sleep disturbance associated with obstructive sleep apnea syndrome, fibromyalgia, chronic pain, and multiple sclerosis and limited evidence for cannabis and HIV-associated cachexia, symptoms of Tourette syndrome, anxiety symptoms (as assessed by a public speaking test, in individuals with social anxiety disorders), and posttraumatic stress disorder (National Academies of Sciences, Engineering, and Medicine, Health and Medicine Division, Board on Population Health and Public Health Practice, Committee on the Health Effects of Marijuana: An Evidence Review and Research Agenda 2017). A more recent systematic review and meta-analysis also reported on potential therapeutic benefit of cannabinoids (McKee et al. 2021). The authors concluded there is limited evidence of the effectiveness of CBD to treat psychiatric symptoms (McKee et al. 2021). Similarly, a systematic review on the effectiveness of medical cannabis for psychiatric, movement and neurodegenerative disorders reported that a definitive conclusion on cannabinoid efficacy could not be drawn (Lim et al. 2017). It reported that upon evaluation of trials, there were significant methodological issues including inadequate description of allocation concealment, blinding, and underpowered sample size (Lim et al. 2017). Another publication included an evidence map of 44 systematic reviews which included evidence from 158 individual studies (Montero-Oleas et al. 2020). Conditions included in the analysis were multiple sclerosis, movement disorders (Tourette’s syndrome, Parkinson disease), psychiatry conditions, Alzheimer’s disease, epilepsy, acute and chronic pain, cancer pain, neuropathic pain, symptoms related to cancer (vomiting, anorexia related to chemotherapy), rheumatic disorders, HIV-related symptoms, glaucoma, and COPD. Evidence was heterogeneous regarding the conclusions and quality of primary studies (Montero-Oleas et al. 2020). Medical cannabis has also been examined for conditions such as gynecologic pain conditions, with most data from surveys of women in prospective cohort studies (Liang et al. 2022). Although most women reported pain reduction, the authors caution that interpretation of data is limited due to varying cannabis formulations, delivery methods, and dosages precluding definitive conclusions (Liang et al. 2022). Taken together, although cannabinoids’ effectiveness has been examined in a wide spectrum of conditions, conclusions are often weak due to important methodological limitations.

Through the Cannabis Act, these changes can be documented and supported under medical supervision. Moreover, to better understand potential harms associated with cannabis use, Health Canada maintains a system for reporting adverse events associated with cannabis products through its Canada Vigilance website (Health Canada 2020a). The government of Canada outlines adverse event responsibilities for both retailer and healthcare providers. Consumers or patients who are using cannabis products for medical or nonmedical purposes and who have experienced a side effect are encouraged to consult with their healthcare practitioner for both management and for completion of side effect reports. This can be done online or via a toll-free telephone number. Guidance on how to complete the report is provided on the website, and additional information can be found at MedEffect Canada (Health Canada 2020b).

The premise of fairness and equity is the backbone of Canada’s healthcare system, which provides universal and publicly funded healthcare. It was created from the Canada Health Act and strives to comply with the five pillars of that act, which states that the system must be universal, publicly administered, have comprehensive coverage, portable across provinces, and accessible to the population. An important limitation of the current Cannabis Act is that decisions about retail cannabis distribution are delegated to individual provinces and territories, resulting in regional heterogeneity in retail approaches. For example, in Quebec, there are government retailers which sell cannabis, whereas in British Columbia, there are mixed public and private retail businesses (Fischer et al. 2021; Wilkins and Rychert 2021). In terms of access, there are inequities across provinces. The Cannabis Act permits direct mail order and home delivery anywhere in Canada for individuals medically authorized to use cannabis. However, this is only available in certain provinces under the Cannabis Act, thereby limiting access to eligible patients. Regulatory variations across provinces contribute to uncertainty of healthcare providers about appropriateness of authorizing medical cannabis, namely cannabidiol (CBD), to pediatric populations for the treatment of epilepsy (Minhas and Lunn 2022). As prescription CBD products are not available in Canada, the Cannabis Act is the only legal mechanism to obtain CBD for disorders such as pediatric epilepsy (Huntsman et al. 2021). Provincial discrepancies have also raised concerns about disparities in cannabis use and harms. Following legalization of recreational cannabis, there has been increased concentrations of cannabis retailers in neighbourhoods of lower socioeconomic status (Myran et al. 2022). The variation has been fuelled by rapid expansion of retail outlets in certain jurisdictions, especially those with a private retail market (Myran et al. 2022).

There are also limitations with the existing medical document which requires the clinician to provide the quantity authorized in grams of dried flower per day. Cannabinoids extracted from dry flower varies, which renders daily dosage conversion from milligrams of the desired cannabinoid to grams of dried cannabis highly variable and complicating the authorization process. Furthermore, different processes for cannabinoid extraction from dried cannabis plant may result in extracts with distinct cannabinoid, terpenoid, and residual solvent profile (Hazekamp 2018). The most appropriate form of medical cannabis depends on the indications for use. Accurate and appropriate prescription of oral oils and capsules requires specifying milligrams of extracted delta-9 tetrahydrocannabinol (THC) and CBD. Furthermore, the medical document does not require specific dosing instructions. Documents should also be revised to allow for the inclusion of more than one product and those administered by different routes (i.e., oil taken orally vs vaporized) on the same form. The most appropriate, accurate, and pragmatic solution is the use of a standard medical prescription document for all clinicians who authorize cannabis.

From a fiscal perspective, there are also important considerations for accessibility to cannabis. The Cannabis Act allows costs for Canadian veterans and other patients, such as those suffering from workplace injury, to be covered by some public or private insurers. This process recognizes cannabis as a medical necessity for these patients. The process of medical authorization of cannabis enables coverage to be determined and maintained as deemed medically appropriate. Additionally, in contrast to other prescription medication, sales and excise tax add further inappropriate financial burden to patients. Most patients pay out of pocket due to lack of insurance coverage. A survey by Health Canada reported that 91% of persons using cannabis for medical reasons reported no insurance coverage (Health Canada 2021). As with all prescription medications, opioids are not taxed and are covered by government and insurance plans; there is concern that patients may choose opioids over cannabis even though they are less safe, on a patient and societal level, for long-term use (Minhas and Lunn 2022).

There are also noteworthy limitations with the Cannabis Act. Despite a regulatory system in place which allows access to cannabis for medical purposes since 2001, regulatory requirements restrict researchers to using exclusively products manufactured under Good Manufacturing Practices (GMP) in human clinical trials. The purpose for this restriction is to ensure products adhere to quality standards of consistency and control (Rueda et al. 2022a). This restriction is problematic since commercial cannabis, purchased and used by patients and consumers in the “real-world” setting, is manufactured under Good Production Practices (GPP). GMP cannabis not being commercially manufactured, renders the product, for the most part unavailable severely impeding research. Since the cannabis industry is not required to partake in the standard drug approval process when seeking approval for medical use, there is little incentive to support research to have their products approved for the medical market (Rueda et al. 2022a). Furthermore, researchers must wait in the same cue as licensed producers when seeking approvals from Health Canada, making for potentially lengthy waiting times and delaying study initiation.

### Is a distinct medical access program necessary to provide individuals with reasonable access to cannabis for medical purposes?

The goals of medical and nonmedical cannabis are very distinct. In our view, access to medical cannabis cannot be ensured through the current nonmedical framework, and these two streams require distinct access streams. Reasons for the separation of the two streams are outlined in Table 1.

**Table 1:** Reasons requiring maintenance of a separate stream for cannabinoid-based medicine (CBM)

• Ensure adequate guidance from healthcare providers (HCPs) to medical cannabis user
• Provide monitoring for adverse events and completion of adverse drug event forms by HCP
• Eliminate stigma experienced by CBM users and providers
• Enable reimbursement of CBM
• Facilitate removal of taxation on CBM
• Promote research on all aspects of CBM
• Ensure adequate supply of medical CBM products

## Patient perspective

Several studies have been conducted on the use of cannabis for the management of diverse types of chronic pain. Research typically demonstrates moderate benefit of cannabis in chronic pain management. There is also evidence for efficacy of cannabis in the management of comorbidities, including sleep disorders, anxiety, and appetite suppression, and for managing symptoms in some chronic conditions associated with pain including HIV, multiple sclerosis, fibromyalgia, and arthritis. However, there exist several challenges within cannabis-based medicines and chronic pain research. With respect to some co-occurring conditions, there still exist relatively few controlled trials. As we reviewed, data related to comorbid conditions, it was not typically the primary focus of included studies and, subsequently, may be underpowered. The lack of comparative studies where the safety and efficacy of cannabis and cannabis-based medicine are compared with typical pain treatments is also problematic. In addition, challenges commonly exist with unmasking in placebo-controlled trials, representing potential risk of bias, especially as pain and many comorbidities are measured with VAS or other subjective measures (Bell et al. 2023).

Importantly, cannabis use may also be associated with both short-term and long-term harms, and these effects have significant inter-patient variability (Health Canada 2023). Short-term risks include drowsiness, slowing of reaction time, reducing ability to pay attention, and impaired coordination, all of which can impact driving or operating equipment. Learning and memory can be adversely impacted. Cannabis can also affect mental health and induce panic and anxiety or trigger a psychotic episode (Health Canada 2023; Siklos-Whillans et al. 2021) In the long-term, inhalation of smoked cannabis is associated with exposure to the same toxic chemicals as found in cigarette smoke. Over time, cannabis use can result in cannabis dependence in the form of addiction, cannabis use disorder, and problematic cannabis use. Addiction can lead to serious harm to health, social life, occupation, and financial future (Health Canada 2023; Siklos-Whillans et al. 2021). Each method of cannabis consumption carries different health and safety risks, and each person’s response is different and depends on sex, age, THC and CBD content, preexisting medical conditions, previous experience with cannabis, frequency of use, and consumption of food, alcohol, or other drugs or health products. Everyone’s response to cannabis may also vary from one time to the next (Health Canada 2023; Siklos-Whillans et al. 2021).

As with all medications, optimal therapy requires clinical guidance to provide appropriate patient selection, safe dosing, method of administration, monitoring of efficacy and tolerability, avoidance of side effects, drug and disease interactions, and patient education (Huntsman et al. 2021; Wright et al. 2020). Pediatric use, usually for management of refractory epilepsy, is an extreme example of the need for a medical stream to ensure not only safety and efficacy but also for access to specific CBD products (Huntsman et al. 2021). Under the law, cannabis treatment for children must be authorized by a physician or nurse practitioners, which provides an opportunity for patient counselling and education (Huntsman et al. 2021). This can only be provided through a medical framework. Patients must not rely on the advice of cannabis salespersons, commonly referred to as “budtenders,” who lack the knowledge, experience, training, and motivation for the task. Additionally, if the medical cannabis stream is removed, then pediatric patients will be required to obtain cannabis from a dispensary. However, currently to purchase recreational cannabis, you must be 19 years of age. Thus, for a pediatric patient to obtain medical cannabis, their parent would need to purchase it under their name and then “divert” the cannabis to the child, which is clearly inappropriate. Another major consideration pertains to the stigma associated with cannabis, which remains a major barrier to use (Troup et al. 2022; Ng et al. 2021). Patients require the support of a healthcare provider not only to manage their own trepidation but also to legitimize use to their family, employer, and broader social network. Moreover, the fact that licensed naturopathic doctors (NDs) are not included in the list of HCPs who can authorize cannabis should be re-examined. NDs are the very HCPs who are specifically trained in botanical medicine and pharmacognosy (the science of natural drugs obtained from organisms such as most plants, microbes, and animals), but they were entirely overlooked in the Cannabis Act. NDs have prescriptive authority in Ontario and British Columbia, and naturopathic medicine is a regulated health profession with government oversight. Therefore, the Cannabis Act may benefit from an amendment to include a regulated health profession that is most familiar with plant-based medicines. Finally, government photo identification is required to purchase cannabis via dispensaries, but not in medical stream. This creates a further barrier for individuals without photo identification, as may be the case for homeless individuals who could benefit from using cannabis as a harm reduction tool.

## The healthcare system

Cost is a major barrier to utilization of medical cannabis. For example, 40 mg of CBD + 10 mg THC daily is approximately US $170 CAD/month. While some insurers and Veteran’s Affairs provide coverage for cannabis accessed through the medical program, it is unlikely to continue in the absence of such a program. Important issues also exist related to taxation. Currently, medical cannabis users in Canada must pay full sales tax. Additionally, federal excise (“sin”) tax is applied to all products containing THC in the amount of US $1 CAD/g or 10% of the final price. Additionally, 4 provinces add provincial/territorial excise (“sin”) tax, including Alberta, Ontario, Saskatchewan, and Nunavut. While this is appropriate for nonmedical use, cannabis is the only prescribed medication to which any taxation applies. We believe this is inappropriate. An Environics poll indicated that 62% of Canadians opposed taxation on medical cannabis, and there is strong patient advocacy to eliminate this tax (Canadians for Fair Access to Medical Marijuana (CFAMM) n.d.). Currently, patients can claim costs of medical cannabis as a deductible medical expense, if approved by a physician, when filing their annual income tax. The absence of a cannabis medical stream will exclude any possibility of elimination of unfair taxation and remove the medical expense deduction. In Canada, prices of legal cannabis are significantly higher than those from illicit sources, largely due to taxation (Health Canada 2022). This disincentivizes use of the safe legal source (Rosic et al. 2021). Therefore, while the Cannabis Act was designed to remove the illicit market, it may actually be strengthening the illicit market when retail prices are too high.

Despite the wide spectrum of opinions regarding the need for distinct medical versus recreational streams, there is broad agreement that there is an urgent need for medical cannabis research. There are many areas of cannabis medicine which require better understanding. In addition to regulatory challenges with conducting research, there are also challenges related to the fact that motives for cannabis use frequently straddle boundaries between medical versus recreational use. For example, in the context of stress management and anxiety, individuals frequently report cannabis use for these indications as both medicinal and recreational (Mannes et al. 2018; Bruce et al. 2020). In a self-report assessment of 709 cannabis users in Canada prior to federal cannabis legalization, Turna et al., characterized patterns of cannabis use (recreational and medical), other substance use, and psychiatric symptoms (Turna et al. 2020). Sixty-one percent of participants endorsed exclusively recreational use, and 39% reported some level of medical use. Compared to recreational users, medical users reported more problematic cannabis use and greater burden of psychiatric symptoms, such as anxiety, depression, and trauma. A large majority of medical users also reported using recreationally, while exclusive medical use was less frequent (81% vs. 19%, respectively) (Turna et al. 2020). Participants in the dual-motives group reported more daily cannabis use and more alcohol and tobacco use. Moreover, participants using cannabis for both medical and recreational purposes more often used cannabis to treat psychiatric conditions compared to participants endorsing medical-only use. Interestingly, several participants reported decreasing their other medications, such as analgesics, anti-inflammatories, and antidepressants in lieu of cannabis (Turna et al. 2020). As the authors highlight, reduction of antidepressant doses may be the cause for concern given that antidepressants are first-line efficacious for anxiety and depression Thus, differences in cannabis use patterns and preferences exist between recreational and medical cannabis users and within medical users. As suggested by the authors, dual motive individuals who use cannabis may warrant special attention as a subpopulation (Turna et al. 2020).

Research funding opportunities are made available, both at the federal level, for example, by the Canadian Institutes for Health Research and various provincial initiatives. The absence of a cannabis medical stream will work strongly against ongoing research support (Rueda et al. 2022b; C. B. C. Radio 2021; Webster 2021). In terms of strain and format availability, under the current 2-stream model, licensed cannabis producers offer a wide variety of medical cannabis products (e.g., flower, oil, capsules) and ranges of CBD, THC, and combined forms. However, nonmedical cannabis users tend to focus on smoked cannabis, with high THC and low CBD levels. The absence of a dedicated medical cannabis stream will likely reduce demand for high CBD and low THC chemotypes in oral formulations. As many licensed producers tend to be fiscally motivated, the absence of a cannabis medial stream may reduce availability of appropriate chemotypes and accurate dosing formats.

## The healthcare provider

Although most trainees had little exposure to cannabis and cannabinoid-based medicine (CBM) in their curricula in the past (Elkrief et al. 2020; St Pierre et al. 2020), most Canadian medical schools have now incorporated CBM into their programs. Accredited continuing health education programs on CBM are widely available. However, the absence of a cannabis medical stream will likely reduce or eliminate the availability of ongoing medical education. Furthermore, stigma directed at CBM prescribers will likely persist, or even worsen, in the absence of a medical cannabis stream (Troup et al. 2022; Ng et al. 2021).

There is controversy within the medical community about the need for separate medical and recreational streams for cannabis (Owens 2018). The Canadian Medical Association does not support the maintenance of separate streams “primarily related to the limited evidence to support many of the therapeutic claims made regarding cannabis for medical purposes, and the need to support health practitioners in their practice” (Canadian Medical Association 2019). In contrast, the Canadian Nurses Association (CNA) statement on medical cannabis is as follows: “The CNA supports and advocates for two streams (medical and non-medical). Supporting these two streams aligns with CNA’s work on access to care and equity” (Canadian Nurses Association n.d.). The Canadian Pharmacists Association statement on medical cannabis indicates that “two distinct streams will allow for the unique needs of medical patients to be met, will contribute to harm reduction, that patients should not be expected to self medicate without support from HCP, it will protect the supply chain to meet patient needs, could lead to more systemic benefits” (Canadian Pharmacists Association 2016). A distinct medical stream for cannabis is also supported by the Arthritis Society of Canada, the Canadian Spondylitis Association, the Canadian Arthritis Patient Alliance, and the Canadian Society of Intestinal Research (GI Society n.d.; Canadian Arthritis Patient Alliance n.d.; Canadian Spondylitis Association n.d.; Arthritis Society of Canada n.d.). Our views are consistent with these latter healthcare associations which are in favor of separate medical and recreational streams.

### What specific reforms are recommended?

We recommend the continuation of a distinct medical cannabis stream, in addition to several adjustments to improve the current systems in place, as outlined in Table 2. New therapeutic potential for cannabinoids is discovered when observations are documented by HCPs and reporting under medical supervision, as provided through the Cannabis Act. Adverse event reporting could take place through the Canada Vigilance Program (Huntsman et al. 2021). Community pharmacies are ideally placed to dispense medical cannabis products, as most already have the necessary infrastructure and experience in place for ordering and storing controlled substances and providing education and ongoing support (Huntsman et al. 2021). Additionally, although it is not standard across the country, some colleges, such as the Ontario College of Pharmacists (Ontario College of Pharmacist n.d.), mandate cannabis education for all pharmacist who are licensed to provide direct patient care by their college. As pharmacists are trained to assess drug-drug interactions and adverse effects, we support the CPHA position statement on medical cannabis which states that pharmacists are best suited to advise patients and oversee the safe storage and dispending of medical cannabis (Huntsman et al. 2021; Canadian Pharmacists Association 2016). Health Canada should also mandate reporting of serious adverse effects associated with medical cannabis by pharmacists and other healthcare providers.

**Table 2:** Recommended reforms for the current medical cannabis stream

• Encourage documentation and reporting by healthcare providers (HCPs), such as when new observations are made regarding the use of medical cannabis
• Mandate adverse event reporting by HCPs through the Canada Vigilance Program
• Increase the involvement of pharmacists in the dispensation of cannabis, screening for drug interactions, and on provision of counseling for medical cannabis
• Encourage colleges of pharmacists to strongly encourage cannabis education for pharmacists
• Mandate reporting of serious adverse effects associated with medical cannabis by pharmacists and other healthcare providers
• Create a simplified authorization process to help clinicians and create a medical document emulating a standard medical “prescription” which specifies a total quantity of each cannabinoid and clear dosing instructions
• Remove the tax on medical cannabis
• Reform the Cannabis Act to permit the use of Good Production Practices (GPP) cannabis clinical research
• Review the quality control regulations of products under the Access to Cannabis for Medical Purposes Regulations (ACMPR) or the Cannabis Act
• Incorporate the consumer health products framework, which will have impact on medical access to cannabidiol (CBD) products and allow the ACMPR to focus on access to delta-9 tetrahydrocannabinol (THC)
• Expand all forms of research, including that on harm reduction and cost-effectiveness of cannabis
• Take into consideration the perspectives of diverse patient representatives prior to implementing changes to the Cannabis Act

From an HCP perspective, we suggest creation of a simplified authorization process to help clinicians (i.e., use specific dosing in mg, not grams of flower/day), and that the medical document emulates a standard medical “prescription” specifying a total quantity of each cannabinoid and clear instruction on dosing, for example, *THC 5 mg capsules taken by mouth twice daily, CBD 20 mg capsules taken by mouth twice daily, dispense 1 month supply*. The tax placed on medical cannabis should be removed.

We urge reform to the act permitting the use of GPP cannabis in clinical research as people who use cannabis for medical purposes currently access commercial products manufactured under GPP standards. We also urge for review the quality control regulations of products under the Cannabis Act, since patients who require close dose monitoring and particular formulations require assurance that their products are manufactured to an acceptable standard of quality. It is necessary to incorporate the consumer health products framework, since this will have impact on medical access to CBD products (enabling pharmacy support) and allow the Cannabis Act to focus on access to THC. Expanding all forms of research, including that on harm reduction and cost-effectiveness of cannabis, since real-world evidence suggests that access to medical cannabis is associated with reduced prescribed medication and healthcare utilization. Individuals using medical cannabis have less opioid use (Boehnke et al. 2016; Reiman et al. 2017). In prospective cohorts in Vancouver of persons at high risk of opioid overdose, consistent cannabis use was associated with increased engagement in opioid agonist treatment for opioid use disorder (Hurd et al. 2019), reduced frequency of illicit opioid use for chronic pain, and lower rates of fentanyl exposure (Lake et al. 2019, 2020). Lastly, we suggest ensuring the perspectives of diverse patient representatives are taken into account prior to implementing any changes to the Cannabis Act. These reforms could substantially improve health-related outcomes as well as effectiveness and cost-effectiveness of many healthcare interventions. Patient engagement with HCP has been associated with positive treatment outcomes including improved control of diabetes, better physical functioning in rheumatic diseases, enhanced patients’ compliance with secondary prevention measures, and improved health in individuals who have suffered myocardial infarction (Arnetz et al. 2004, 2008; Rachmani et al. 2002; Loh et al. 2007). In fact, patient participation is not only regarded as a legal right of patients but is also considered an international gold standard for healthcare systems (Vahdat et al. 2014).

## Data Availability

Not applicable.

## Abbreviations

CBD: Cannabidiol
ACMPR: Access to Cannabis for Medical Purposes Regulations
CNA: Canadian Nurses Association
GMP: Good manufacturing practices
GPP: Good Production Practices
HCP: Healthcare provider
MMPR: Marijuana for Medical Purposes Regulations
NDs: Naturopathic doctors
THC: Delta-9 tetrahydrocannabinol
